# Poor perfusion of the microvasculature in peritoneal metastases of ovarian cancer

**DOI:** 10.1007/s10585-020-10024-4

**Published:** 2020-02-01

**Authors:** Arnoud W. Kastelein, Laura M. C. Vos, Juliette O. A. M. van Baal, Jasper J. Koning, Vashendriya V. V. Hira, Rienk Nieuwland, Willemien J. van Driel, Zühre Uz, Thomas M. van Gulik, Jacco van Rheenen, Can Ince, Jan-Paul W. R. Roovers, Cornelis J. F. van Noorden, Christianne A. R. Lok

**Affiliations:** 1 Department of Obstetrics and Gynecology, University of Amsterdam, Amsterdam UMC, Location AMC, Amsterdam, The Netherlands; 2 Center for Gynecologic Oncology Amsterdam, Department of Gynecologic Oncology, The Netherlands Cancer Institute/Antoni Van Leeuwenhoek Hospital, Amsterdam, The Netherlands; 3 Department of Molecular Cell Biology and Immunology, Free University, Amsterdam UMC, Location VUMC, Amsterdam, The Netherlands; 4 Cancer Center Amsterdam, Department of Medical Biology, University of Amsterdam, Amsterdam UMC, Location AMC, Amsterdam, The Netherlands; 5 Department of Genetic Toxicology and Cancer Biology, National Institute of Biology, Ljubljana, Slovenia; 6 Laboratory of Experimental Clinical Chemistry and Vesicle Observation Centre, University of Amsterdam, Amsterdam UMC, Location AMC, Amsterdam, The Netherlands; 7 Department of Surgery, University of Amsterdam, Amsterdam UMC, Location AMC, Amsterdam, The Netherlands; 8 Department of Molecular Pathology, The Netherlands Cancer Institute and Oncode Institute, Amsterdam, The Netherlands; 9 Department of Translational Physiology, University of Amsterdam, Amsterdam UMC, Location AMC, Amsterdam, The Netherlands; 10 Department of Intensive Care, Erasmus University, Erasmus MC, Rotterdam, The Netherlands; 11 Department of Obstetrics and Gynecology, University of Amsterdam, Amsterdam UMC, Location AMC, Meibergdreef 9, Room H4-240, 1105 AZ Amsterdam, The Netherlands

**Keywords:** Microvasculature, Microcirculation, EOC, Peritoneal carcinomatosa, Incident dark field imaging

## Abstract

Most women with epithelial ovarian cancer (EOC) suffer from peritoneal carcinomatosis upon first clinical presentation. Extensive peritoneal carcinomatosis has a poor prognosis and its pathophysiology is not well understood. Although treatment with systemic intravenous chemotherapy is often initially successful, peritoneal recurrences occur regularly. We hypothesized that insufficient or poorly-perfused microvasculature may impair the therapeutic efficacy of systemic intravenous chemotherapy but may also limit expansive and invasive growth characteristic of peritoneal EOC metastases. In 23 patients with advanced EOC or suspicion thereof, we determined the angioarchitecture and perfusion of the microvasculature in peritoneum and in peritoneal metastases using incident dark field (IDF) imaging. Additionally, we performed immunohistochemical analysis and 3-dimensional (3D) whole tumor imaging using light sheet fluorescence microscopy of IDF-imaged tissue sites. In all metastases, microvasculature was present but the angioarchitecture was chaotic and the vessel density and perfusion of vessels was significantly lower than in unaffected peritoneum. Immunohistochemical analysis showed expression of vascular endothelial growth factor and hypoxia inducible factor 1α, and 3D imaging demonstrated vascular continuity between metastases and the vascular network of the peritoneum beneath the elastic lamina of the peritoneum. We conclude that perfusion of the microvasculature within metastases is limited, which may cause hypoxia, affect the behavior of EOC metastases on the peritoneum and limit the response of EOC metastases to systemic treatment.

**Electronic supplementary material:**

The online version of this article (10.1007/s10585-020-10024-4) contains supplementary material, which is available to authorized users.

## Introduction

Epithelial ovarian cancer (EOC) has the highest mortality of all gynecological malignancies. Worldwide, an estimated 140,000 women die annually from this type of cancer [1, 2]. At the time of first clinical presentation, over 70% of women with EOC have advanced stage disease that has spread beyond the pelvis to the peritoneum of the abdominal cavity. This peritoneal carcinomatosis (PC) produces malignant ascites and impairs bowel movements, which has a severe impact on quality of life and survival [3, 4].

PC poses a challenge for clinicians and researchers. Complete cytoreductive surgery is difficult, especially when the small bowel is affected by PC. Although systemic intravenously (IV)-administered chemotherapy is often initially effective, recurrences occur in more than 60% of women [3, 5]. Moreover, interactions between metastatic EOC cells and the peritoneum are still an enigma: it is unclear why peritoneal metastases of EOC remain small (1–2 mm^3^) and why spontaneous invasive growth of EOC cells through the parietal peritoneal layers does not occur [6-8].

Formation of new blood vessels (angiogenesis) and a new vascular network is required for tumors to grow larger than 1–2 mm^3^ and is considered a prerequisite for tumor progression [9-13]. Previous immunohistochemical studies reported increased vascularization in metastases and the surrounding peritoneum [14-16]. However, the perfusion of the microvasculature was not investigated in these ex vivo studies and it has not been clearly described whether there is vascular continuity between peritoneal metastases and the vascular network of the submesothelial stroma of the peritoneum [6].

Incident dark field (IDF) imaging enables real-time in vivo visualization of the human microvasculature, allowing quantification of perfusion [17]. We recently visualized the peritoneal microvasculature with IDF imaging and we showed that the peritoneal microvasculature consists of well-perfused vessels forming an organized network, often embedded in fat tissue [18].

We hypothesized that the microvasculature in peritoneal EOC metastases is insufficient, which may not only limit expansive and invasive growth, but also impair the therapeutic efficacy of systemic IV chemotherapy. Therefore, the present study aimed to investigate the angioarchitecture and perfusion of the microvasculature in peritoneum and peritoneal metastases of EOC. We performed IDF imaging in patients with and without EOC and/or PC and we performed histology and immunohistochemistry on tissue derived from IDF-imaged sites. Three-dimensional (3D) whole tumor imaging was performed using light sheet fluorescence microscopy, to allow a circumferential analysis of metastases and to investigate whether the microvasculature of metastases is continuous with that of the surrounding submesothelial stroma.

## Materials and methods

### Participants

In this prospective single-center observational study, we recruited women undergoing either cytoreductive surgery for advanced EOC or exploratory laparotomy because of a suspicious adnexal mass. This study was performed at the Department of Gynecological Oncology of the Netherlands Cancer Institute, Antoni van Leeuwenhoek Hospital, Amsterdam, The Netherlands. Participant characteristics were retrieved from medical charts. Exclusion criteria were recent cardiovascular events, use of anticoagulant or immunosuppressant medication and language barriers compromising informed consent. Participants that were treated with neoadjuvant chemotherapy were not excluded. The study was in compliance with ethical principles and regulatory requirements according to the Declaration of Helsinki. All study participants received a verbal and written explanation of the study procedures. Informed consent was obtained from all individual participants included in the study. The protocol was reviewed by the Institutional Review Board of the Antoni van Leeuwenhoek Hospital and permission was granted on August 16, 2017.

### Vital parameters

Parameters such as heart rate, blood pressure, body temperature, hemoglobin levels and hematocrit were acquired at the time of microcirculatory imaging. An hypotensive event was characterized as a drop of > 20% in mean arterial pressure or a mean arterial pressure of < 60 mmHg [19]. Use of inotropic medicine was recorded as total quantity of administered drug until time of imaging.

### Microcirculatory imaging

The microcirculation was visualized with IDF imaging (CytoCam; Braedius Medical, Huizen, The Netherlands) [20]. The CytoCam is a third-generation hand-held video microscope which weighs 120 g and is shaped like a pen (length 220 mm, diameter 23 mm). The CytoCam produces green light (530 nm) produced by concentrically placed light-emitting diodes (LEDs), providing epi-illumination of the tissue. The green light is absorbed by hemoglobin in erythrocytes, allowing erythrocytes to be imaged and recorded as a real-time representation of the local microcirculation [21]. The CytoCam uses a short illumination pulse time (2 ms) and a high spatial (14 megapixels) and temporal (60 frames per second) resolution. A total field of view of 1.55 × 1.16 mm was recorded at a × 4 optical magnification. The system provides an optical resolution of more than 300 lines/mm. The CytoCam is connected to a device controller which in turn is connected to a laptop, to record and store video clips as digital audio video interleave (AVI) files [20].

### Data acquisition

Microcirculatory imaging was performed during surgery as soon as the surgical retractor (Omni-Tract Surgical, St. Paul, MN, USA) was positioned, either before cytoreductive surgery or after removal of the enlarged adnexa. The latter was sometimes necessary to obtain clear vision and access to the pelvic peritoneum. During imaging, the CytoCam was covered with a sterile latex-free probe cover (Microtek Medical, Zutphen, The Netherlands). The camera was operated by trained researchers (AWK, LMCV or CARL). Excess peritoneal fluid was removed using gauze or suction. To prevent pressure-induced artefacts, the camera was placed in light contact with the peritoneal surface in a perpendicular position. In case of a macroscopically normal aspect of the peritoneum, a total of 6 video clips were recorded on specific locations: (1) the paracolic gutter left and right, (2) the ovarian fossa left and right, (3) on top of the bladder left and right. In participants with PC, video clips were recorded by placing the CytoCam directly on top of metastatic depositions, and adjacent to the tumors (< 1–2 cm). All imaged metastases had a diameter ≥ 1 mm. Aforementioned specific locations of the peritoneum were also imaged in participants with PC. At each area of interest, one recording of 3 s was made, unless imaging was suboptimal (e.g. due to image drifting or loss of focus; in those cases, a second or even third recording was performed).

### Quality score

The quality of each video clip was assessed using a scoring system based on 6 parameters (illumination, focus, content, stability, pressure, duration) [22]. When not all 6 parameters scored positively, the recording was not analyzed.

### Quantification of the microcirculation and comparison of microcirculatory parameters

The microcirculation was quantified in the capillaries only, since these are the (functional) exchange vessels [23]. We considered any vessel with a diameter of < 20 µm to be a capillary, which is also the general consensus in the literature [23-25]. We calculated the total vessel density (TVD, mm/mm^2^) and the quality of the blood flow in the capillaries (0, no flow; 1, sluggish flow; 2, intermittent flow; 3, continuous flow). Subsequently, the perfused vessel density (PVD, mm/mm^2^) was determined and the percentage of perfused vessels (PPV, %) was calculated as ratio of all vessels with flow (score ≥ 1) and TVD.

### Image analysis

Analysis of images was performed offline. The analyst was blinded for the macroscopic aspects of the imaged sites. Above-mentioned parameters PVD, PPV and TVD were calculated with the use of software-assisted analysis (AVA 3.2; Automated Vascular Analysis, Amsterdam UMC, location AMC, Amsterdam, The Netherlands).

### Statistical analysis

Descriptive statistics were used to analyze the demographic variables. Non-normally distributed data were presented as medians with interquartile ranges. The non-parametric Mann–Whitney U test was used for comparing medians of TVD, PVD and PPV across the different non-normally distributed groups. Normally-distributed data were presented as means with standard deviations. A two-sided P value < 0.05 was considered to indicate statistically significant differences. All data were analyzed using SPSS, version 23.0 for Windows (IBM, Armonk, NY, USA). We did not perform a power calculation prior to the study, since this was a first pilot study with unknown effect sizes. We aimed to include at least 10 participants with PC. However, because it was not always known prior to inclusion whether PC was present, it was unknown how many participants had to be included to reach the target of 10 participants with PC.

### Histology

Biopsies were taken from metastases and from peritoneum with a normal aspect (control), all from different patients. Prior to resection of the biopsies, their localization was imaged with the CytoCam. Tissue samples were fixed using buffered 4% para-formaldehyde immediately after harvesting. Samples were either used for (1) immunohistochemistry and conventional 2D microscopy or (2) 3D whole tumor imaging using light sheet fluorescence microscopy.

#### Immunohistochemistry

Chromogenic immunohistochemistry was performed on 5 µm-thick paraffin sections of the tissue samples using Benchmark ULTRA staining (Roche Diagnostics, Indianapolis, MN, USA), using the method that was recently shown to be the optimum immunohistochemical method to be applied to paraffin sections [26]. Dewaxing was performed by incubation of the sections in xylene (VWR Chemicals, Atlanta, GA, USA) for 10 min and rinsing in 100% ethanol (Merck, Darmstadt, Germany). Sections were treated with 100% methanol (Merck) containing 0.3% hydrogen peroxide (Merck) for 10 min to block endogenous peroxidase activity and to reduce non-specific background staining, followed by a washing step in distilled water. Antigen-retrieval was performed using Tris–EDTA buffer (pH 8.5) followed by a washing step in distilled water and 3 washing steps of 5 min each using Tris-buffered saline (TBS; pH 7.6) containing 0.1% Triton-X (Sigma-Aldrich, St. Louis, MO, USA). Sections were encircled with a PAP pen (Dako, Glostrup, Denmark) and incubated using TBS containing 3% normal goat serum (Dako) and 0.1% Triton-X for 1 h to further reduce non-specific background staining and for permeabilization of the sections. Sections were subsequently incubated overnight at 4 °C with primary antibodies against paired-box gene 8 (PAX-8 363M-15; clone MRQ50, dilution 1:50; Cellmarque, Rocklin, CA, USA), which specifically stains EOC cells, VEGF (sc-152; Santa Cruz, Biotechnology, Dallas, TX, USA), cluster of differentiation 31 (CD31; M0823, clone JC70A, dilution 1:250, Dako), which specifically stains endothelial cells [26-29]. Next, sections were incubated with 3,3′-diaminobenzidine (Dako) for 10 min, followed by one washing step with tap water to stop the peroxidase enzyme reaction. Sections were then incubated for 30 s in hematoxylin (Sigma-Aldrich) for nuclear counterstaining. Sections were again placed in running tap water for 5 min and then in distilled water. Finally, sections were dehydrated by subsequently dipping in 70%, 96% and 100% ethanol (Merck) and 3 times dipping in xylene (VWR Chemicals). All incubations were performed at room temperature (RT) unless stated otherwise. An amplification step with a secondary horseradish peroxidase (HRP) antibody (poly-HRP) was performed on the PAX-8-stained sections. Finally, sections were covered with the synthetic mountant Pertex (Histolab, Götenburg, Sweden).

Fluorescence immunohistochemical staining of HIF-1α was performed, following the method as described by Hira et al. [30]. Paraffin sections were dewaxed by incubation of the sections in xylene (VWR) for 5 min, followed by rinsing in 100%, 96% and 70% ethanol (Merck), respectively. Antigen-retrieval was performed in a microwave using 100 mM citrate buffer containing 0.1% Triton-X, pH 6.0, for 20 min at 98 °C, followed by cooling for 20 min and a washing step in PBS. Sections were encircled with a PAP pen (Dako) and incubated with PBS containing 10% normal goat serum (Dako) and 0.1% Triton-X for 1 h at RT, to reduce non-specific background staining and for permeabilization of the sections. Sections were subsequently incubated overnight at 4 °C with primary antibody (mouse anti-human HIF-1α, Abcam (ab8366), dilution 1:100) diluted in PBS containing 1% BSA. Sections were washed 3 times using PBS containing 1% BSA. Alexa Fluor 546-conjugated goat anti-mouse antibodies were used as secondary antibodies in a dilution of 1:200 in PBS containing 1% BSA for 1 h at RT. Next, sections were washed in PBS for 5 min and coverslipped using Prolong Gold mounting medium (Life Technologies, Carlsbad, CA, USA). Afterwards, sections were sealed with nail polish and dried overnight.

Control incubations were performed in the absence of the primary antibody. Chromogenic stained sections were analyzed by light microscopy (Olympus BX51 microscope, Leiderdorp, The Netherlands) and images were acquired using the Olympus cellSense Standard software. Fluorescence imaging was performed using a Nikon Eclipse Ti-E inverted microscope (Nikon Instruments, Melville, NY, USA) and the Nikon NIS-Elements AR 4.13.04 software.

#### 3D whole tumor imaging using light sheet fluorescence microscopy

For 3D imaging, samples were treated according to the iDisco protocol with minor adaptions [31]. Samples were dehydrated with methanol/H_2_O series: 20%, 40%, 60%, 80%, 100%, 100%; 1 h each and were incubated overnight in 66% dichloromethane (DCM)/33% methanol at RT under continuous slow shaking. Samples were then washed twice in 100% methanol at RT, subsequently chilled at 4 °C and overnight bleached in fresh 5% H_2_O_2_ in methanol. Next day, samples were rehydrated with methanol/H_2_O series: 80%, 60%, 40%, 20%, PBS; 1 h each at RT and subsequently washed 2 × for 1 h in PBS + 0.2% Triton X-100 at RT. For immunolabeling, samples were incubated for 2 days in permeabilization solution (PBS, 0.4% Triton X-100, 20% DMSO, 0.3 M glycine) at 37 °C and subsequently blocked in blocking solution (PBS/0.2% Triton X-100/10% DMSO/6% donkey serum) for 1 day at 37 °C. Samples were washed in PBS/0.2% Tween-20 with 10 μg/ml heparin (PTwH) for 1 h twice, then incubated in mouse anti-human CD31 antibodies (clone EN4; dilution 1:200; Monosan; Sanbio; Uden) in PTwH/5% DMSO/3% donkey serum at 37 °C for 4 days under continuous shaking. Samples were washed with PTwH for 10 min, 15 min, 30 min, 1 h and every 2 h until the end of the day and incubated with secondary antibody in PTwH, 3% donkey serum for 4 days at 37°, under continuous slow shaking. Samples were washed in PTwH for 10 min, 15 min, 30 min, 1 h, every 2 h and overnight at RT. After immunolabeling, samples were embedded in 2% low melting agarose (Sigma-Aldrich) to facilitate handling of the samples and dehydrated in methanol/H_2_O series: 20%, 40%, 60%, 80%, 100%, 1 h each at RT. Samples were left overnight in 100% methanol and the next day incubated for 3 h in 66% DCM/33% methanol at RT, 2 × in 100% DCM (Sigma) for 15 min and finally incubated in dibenzyl ether (Sigma) (no shaking) until samples were transparent. Upon clearing, samples were immediately imaged with light sheet fluorescence microscopy using the Ultramicroscope (LaVision BioTec, Bielefeld, Germany) [28]. Inspector Software (LaVision, BioTec) was used for settings adjustment and image acquisition. Image analysis was performed using Imaris 9.2 or higher [28].

## Results

Twenty-three participants were included in the study. Participant and disease characteristics as well as perioperative parameters that may have affected the peritoneal microcirculation are summarized in Table 1.

**Table 1:** Characteristics and perioperative parameters of the participants and the disease state

	All participants (n = 23)	Benign (n = 3)	EOC (n = 10)	PC of EOC (n = 10)
Participant characteristics				
Age (years)	69 (58–75)	69 (68–x)	60 (50–72)	66 (71–80)
Smoking, n (%)	3 (13.0)	1 (33.3)	2.5 (0–21.3)	0 (0–11)
Diabetes, n (%)	4 (17.3)	1 (33.3)	0 (0)	3 (30)
Hypertension, n (%)	5 (21.7)	0 (0)	2 (20)	3 (30)
Cardiovascular event, n (%)	8 (34.8)	2 (66.7)	1 (10)	5 (50)
History abdominal surgery, n (%)	15 (65.2)	2 (66.7)	8 (80)	5 (50)
Disease characteristics				
Histotype				
Serous, *n* (%)	15 (65.2)	0 (0)	7 (70)	8 (80)
Clear-cell, *n* (%)	1 (4.3)	0 (0)	1 (10)	0 (0)
Granulosa-cell, *n* (%)	1 (4.3)	0 (0)	1 (10)	0 (0)
Mucinous, *n* (%)	1 (4.3)	0 (0)	1 (10)	1 (10)
Endometrioid, *n* (%)	1 (4.3)	0 (0)	0 (0)	0 (0)
Unknown PAX8-positive, *n* (%)	1 (4.3)	0 (0)	1 (10)	1 (10)
Grade, high grade, n (%)	14 (60.9)	0 (0)	6 (60)	8 (80)
FIGO stage				
Ic, *n* (%)	4 (17.4)	0 (0)	4 (40)	0 (0)
IIb, *n* (%)	1 (4.3)	0 (0)	0 (0)	1 (10)
IIIc, *n* (%)	9 (39.1)	0 (0)	2 (20)	7 (70)
Iva, *n* (%)	3 (13.0)	0 (0)	1 (10)	2 (20)
IVb, *n* (%)	3 (13.0)	0 (0)	3 (30)	0 (0)
Peritoneal involvement, n (%)	10 (43.5)	0 (0)	0 (0)	10 (100)
Simplified PCI	3 (0–6.75)	0 (0)	3 (0–3)	7 (5.3–11.5)
Regionscore	1.5 (0–3.75)	0 (0)	1 (0–2)	5 (3–6.3)
Chemotherapy, n (%)	15 (65.2)	0 (0)	7 (70)	8 (80)
Surgical characteristics				
Explorative, n (%)	5 (21.7)	3 (100)	2 (20)	0 (0)
CR post chemotherapy, n (%)	14 (60.9)	0 (0)	6 (60)	8 (80)
CR in recurrent disease, n (%)	4 (17.3)	0 (0)	2 (20)	2 (20)
Anesthesia				
Intravenous and epidural, n (%)	23 (100)	3 (100)	10 (100)	10 (100)
Hypotensive event, n (%)	9 (39.1)	0 (0)	5 (50)	4 (40)
Norepinephrine (mg)	0.3 (0.2–0.4)	0.3 (0.3–x)	0.2 (0.15–0.3)	0.4 (0.2–1.0)
Ephedrine (mg)	10 (0–20.0)	0 (0)	10 (0–21.3)	12.5 (0–21.3)
Per-operative parameters				
Heart rate (bpm)	64 (57–71)	62 (57–x)	65 (56.3–73.8)	64.5 (55–93)
Systolic pressure (mmHg)	105 (81–130)	116 (66–x)	93 (77.8–137)	107 (90–125)
Diastolic pressure (mmHg)	55 (44–70)	67 (38–x)	54.5 (44.3–76)	54 (44–66)
Mean pressure (mmHg)	69 (54–84)	82 (49–x)	65.5 (50–76)	70 (60–79)
Core temperature, ºC	36.1 (35.7–36.1)	35.9 (35.4–x)	36.1 (35.6–36.6)	36.1 (35.9–36.4)
Hemoglobin (mmol/L)	7.4 (6.8–7.9)	8.1 (7.2–x)	7.7 (7.0–8.0)	6.9 (6.6–7.8)
Hematocrit (fraction)	0.35 (0.32–0.39)	0.39 (0.36–x)	0.35 (0.34–0.40)	0.33 (0.31–0.37)

Data are presented as medians and interquartile ranges unless indicated otherwise

*EOC* epithelial ovarian cancer, *PC* peritoneal carcinomatosis, *FIGO* international federation of Gynecology and Obstetrics, *CR* cytoreductive, *PCI* peritoneal cancer index, *PAX8* paired box gene 8, *X* upper interquartile range value was not available

A total of 199 video clips were recorded, of which 118 videos were accepted for analysis after quality assessment. Exclusion of videos most often occurred because of insufficient stability [32]. Based on histopathology of the tumor and macroscopic aspects of the peritoneum, participants were divided into 3 groups: benign (n = 3, 17 video clips), EOC with no visible PC (n = 10, 55 video clips) and EOC with visible PC (n = 10, 46 video clips).

### IDF imaging: qualitative analysis of angioarchitecture

**Fig. 1:**
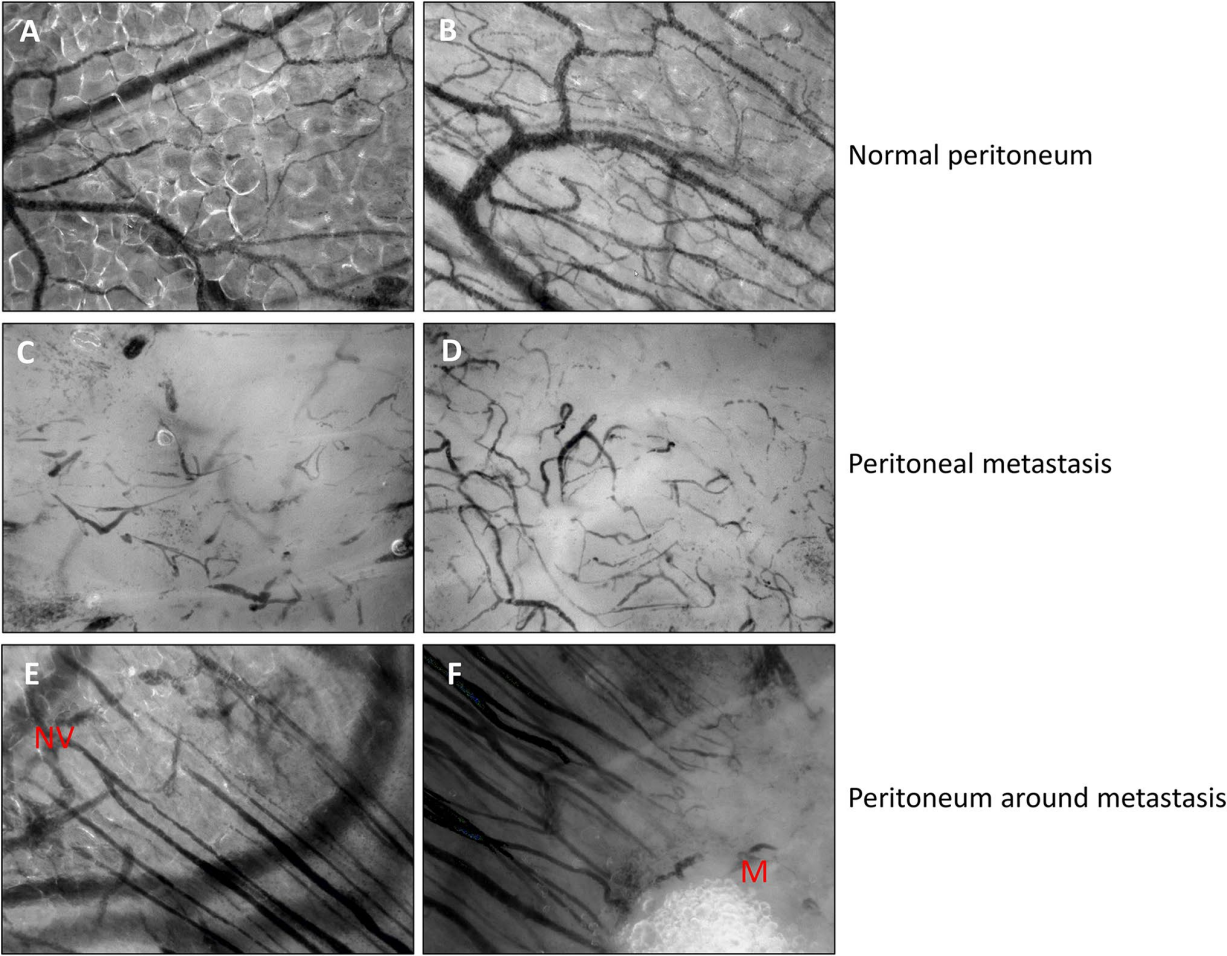
Screenshots from CytoCam—incident dark field imaging of the peritoneal microcirculation. Each image represents an imaged area of 1.55 × 1.16 mm. **A**, **B** The microcirculation (black) in peritoneum with a macroscopically normal aspect showing continuous blood flow. The vessels were embedded in fat tissue and the angioarchitecture was characterized by an organized network of capillaries. **C**, **D** The microcirculation within a peritoneal metastasis of EOC. The angioarchitecture was distinctly different from that in unaffected peritoneum. The blood flow in the microvasculature was either sluggish or absent. **E**, **F** The microcirculation surrounding a peritoneal metastasis. **E** Normal peritoneal microvasculature is indicated by NV. Parallel-aligned capillaries originate from the normal microvasculature and are directed towards a metastasis. **F** Parallel-aligned capillaries that originate from the normal vasculature are directed towards a metastasis (M). Red line, boundary of the metastasis

IDF imaging of the microvasculature revealed extensive differences between the microvascular networks in peritoneum and peritoneal metastases (Fig. 1A–D). The angioarchitecture in peritoneum without peritoneal metastases was characterized by an organized network structure and was comparable in all groups (benign, EOC without PC and EOC with PC) (Fig. 1A, B). In all peritoneal metastases, a disorganized network of heterogeneous capillaries was observed (Fig. 1C, D). The microvasculature surrounding metastases showed functional, parallel-aligned capillaries, that originated from the capillaries of normal peritoneum and were directed towards the metastases (Fig. 1E, F).

### IDF imaging: quantitative analysis of microvascular parameters

**Fig. 2:**
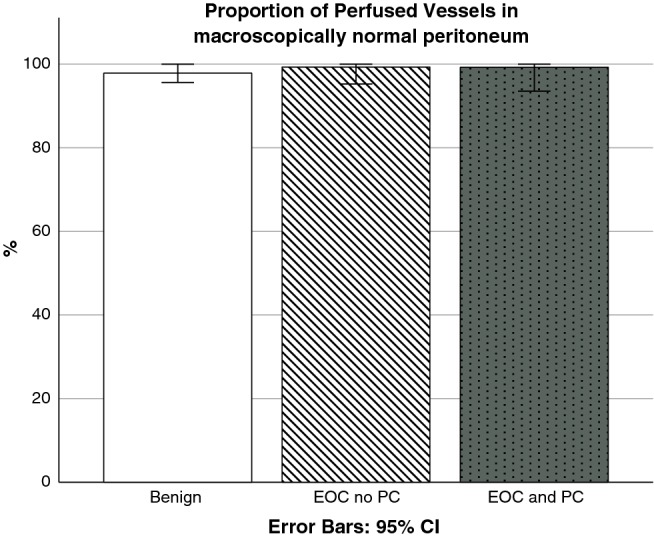
Proportion of perfused vessels in peritoneum with a macroscopically normal aspect in three groups of patients: 1. benign tumor (n = 17 videos), 2. EOC without PC (n = 55 videos) and 3. EOC with PC (n = 32 videos). *EOC* epithelial ovarian cancer, PC peritoneal carcinomatosis

**Fig. 3:**
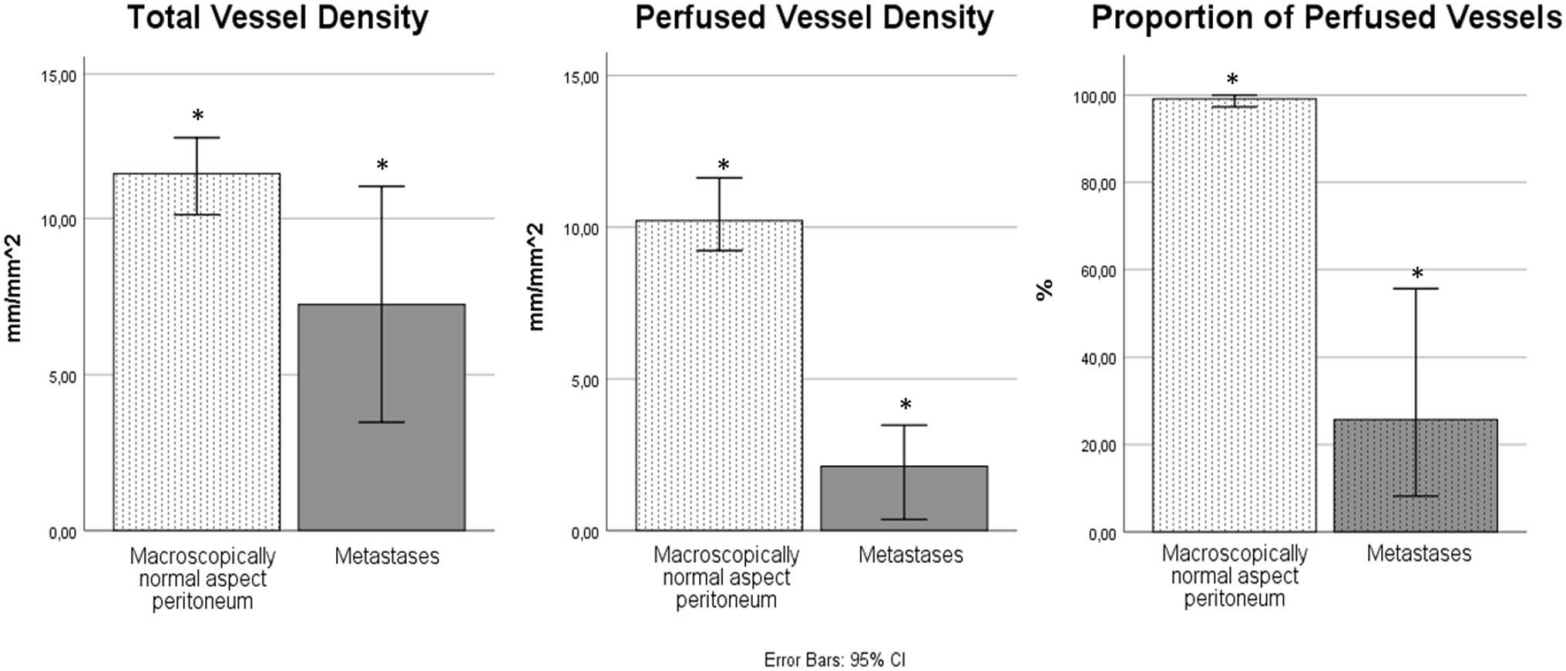
The microvascular parameters total vessel density, perfused vessel density and proportion of perfused vessels in peritoneum with a macroscopically normal aspect (n = 104) compared to peritoneal metastases (n = 14). Significant differences were observed for the perfusion parameters and are indicated with *

Video clips of the microvasculature in peritoneum with a macroscopically normal aspect and of the microvasculature in peritoneal metastases are shown in the animation (Online Resource 1). Quantitative microvascular parameters are shown in Table 2. In peritoneum with a macroscopically-normal aspect, the microvasculature was characterized by a high perfusion rate, irrespective the presence of EOC and/or PC (Fig. 2). In contrast, perfusion parameters (PPV and PVD) and TVD were significantly lower in metastases than in peritoneum with a macroscopically-normal aspect (Table 2; Fig. 3). No differences in microvascular parameters were observed between imaging during primary cytoreductive surgery or after neo-adjuvant chemotherapy.

**Table 2:** Quantitative microvascular parameters

	Benign	EOC, no PC	EOC, PC Macroscopically normal aspect peritoneum	EOC, PC Metastases	P value
Videos, n	17	55	32	14	
PPV (%)	97.9 (95.3–100)	99.3 (90.8–100)	99.3^a^ (92.3–100)	**25.7**^a^**(17.8**–**50.1)**	< 0.01
PVD (mm/mm^2^)	7.9 (6.6–15.7)	11.4 (8.2–13.6)	9.8 ^a^ (7.5–13.8)	**2.1**^a^**(0.5**–**3.2)**	< 0.01
TVD (mm/mm^2^)	8.2 (7.0–17.6)	12.6 (9.5–14.0)	10.6 ^a^ (8.4–13.9)	**7.2**^a^**(3.7**–**10.7)**	0.05

Data are presented as medians and ranges between brackets

*EOC* epithelial ovarian cancer, *PC* peritoneal carcinomatosis, *PPV* percentage of perfused vessels, *PVD* perfused vessel density, *TVD* total vessel density

^a^Parameters were compared

### Immunohistochemistry

**Fig. 4:**
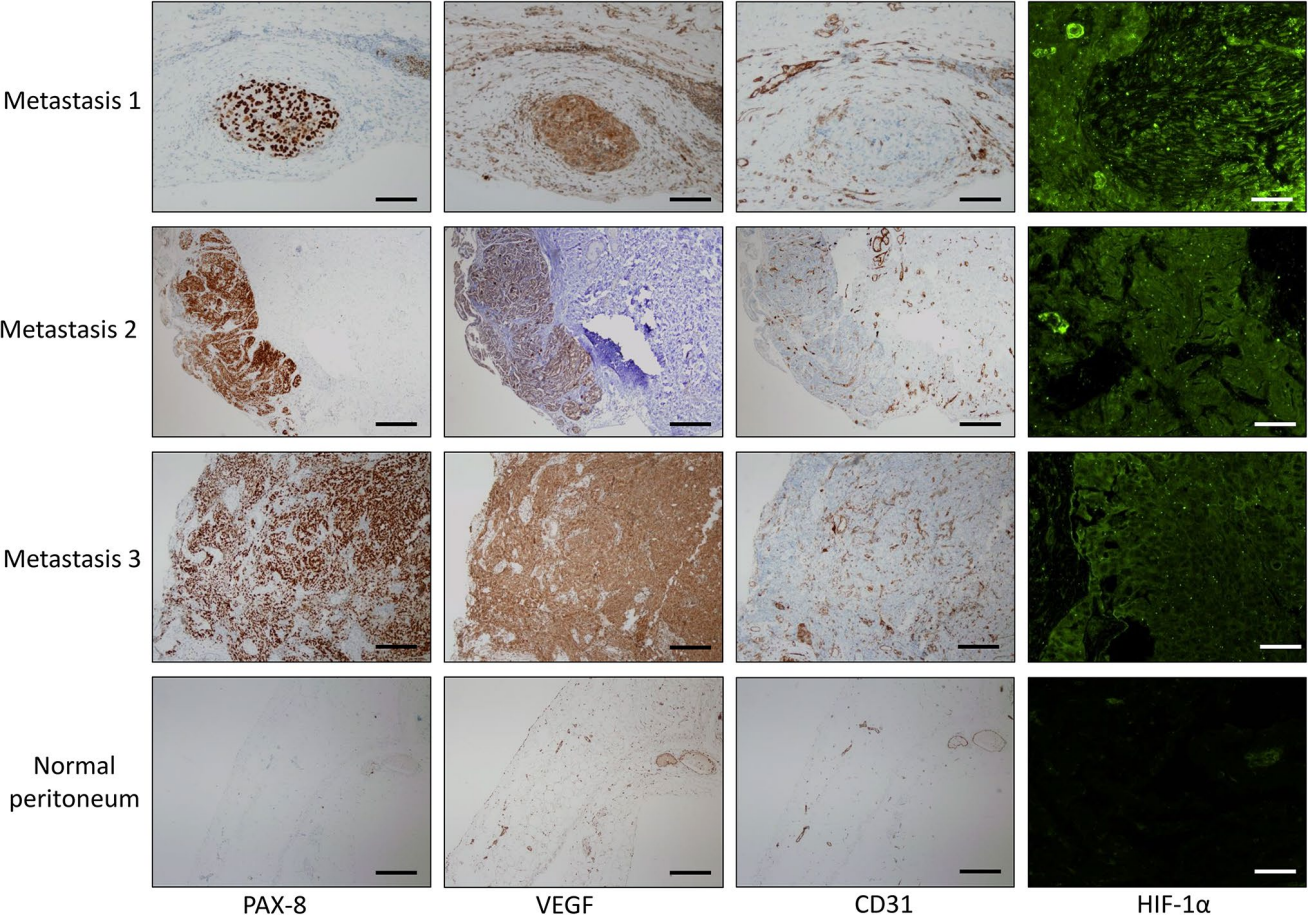
Immunohistochemical (IHC) staining of PAX8, VEGF, CD31 and HIF-1α was performed on paraffin sections of 3 IDF-imaged peritoneal high grade serous carcinoma metastases (metastases 1, 2 and 3). Chromogenic IHC-images of PAX8, VEGF and CD31, black bars = 100 µm. Fluorescence IHC-images of HIF-1α of an area that stained positive for PAX8, white bars = 25 µm. Abbreviations: PAX8, paired box gene 8; VEGF, vascular endothelial growth factor; CD31, cluster of differentiation 31; HIF-1α, hypoxia inducible factor 1α

In three different patients with high grade serous carcinoma (HGSC), imaged tissue sites that were macroscopically determined to contain EOC metastases were biopsied, sectioned and stained (Fig. 4). EOC cells were stained specifically with anti-PAX8 antibodies [27]. All metastases indeed stained for PAX8 protein and all 3 metastases displayed high levels of VEGF, CD31-positive endothelial cells and expression of HIF-1α (Fig. 4; metastases 1, 2 and 3). In all 3 metastases that had been biopsied, IDF imaging showed limited perfusion of the microvasculature. The control sample (peritoneum of a patient with a benign ovarian tumor) did not stain for PAX8. Limited staining for VEGF, CD31 and HIF-1α was observed, especially in the endothelium of peritoneal microvessels (Fig. 4; normal peritoneum).

### Whole mount 3D light sheet fluorescence microscopy

**Fig. 5:**
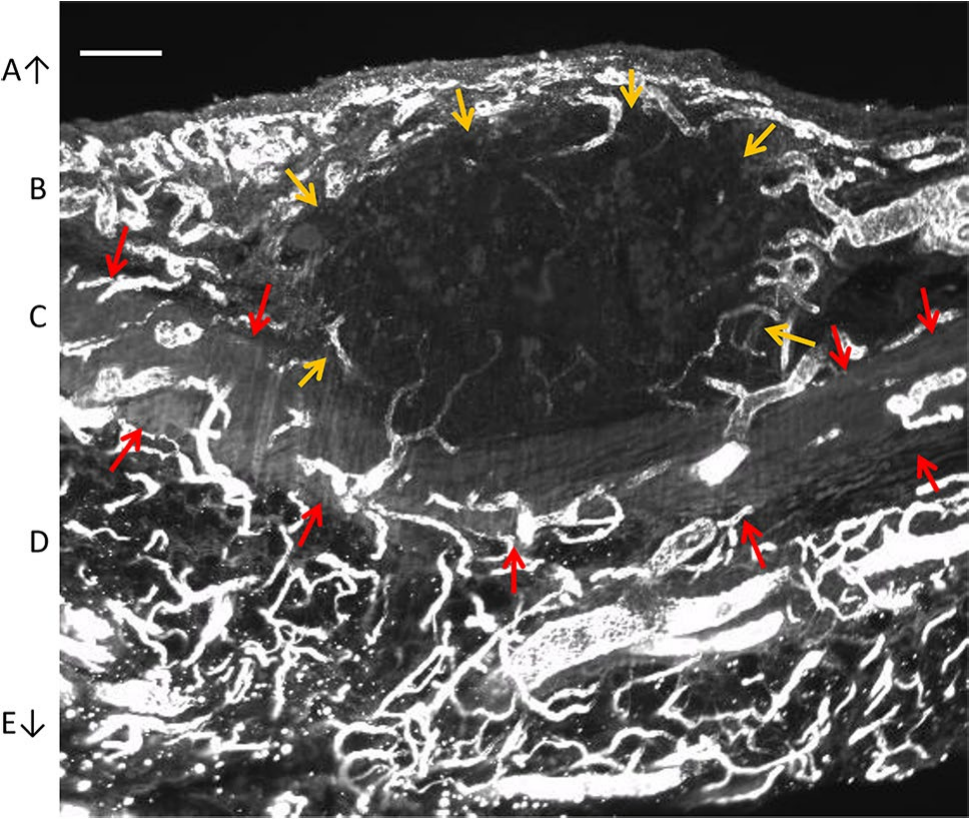
Light sheet fluorescence microscopy image of a peritoneal metastasis with CD31 staining. Blood vessels are shown in white. Boundaries of the metastasis are indicated by yellow arrows, and the boundaries of the elastic lamina (**C**) by red arrows. **A** Abdominal cavity, **B** submesothelial stroma, **C** elastic lamina, **D** submesothelial stroma; **E** abdominal wall. Bar = 200 µm

**Fig. 6:**
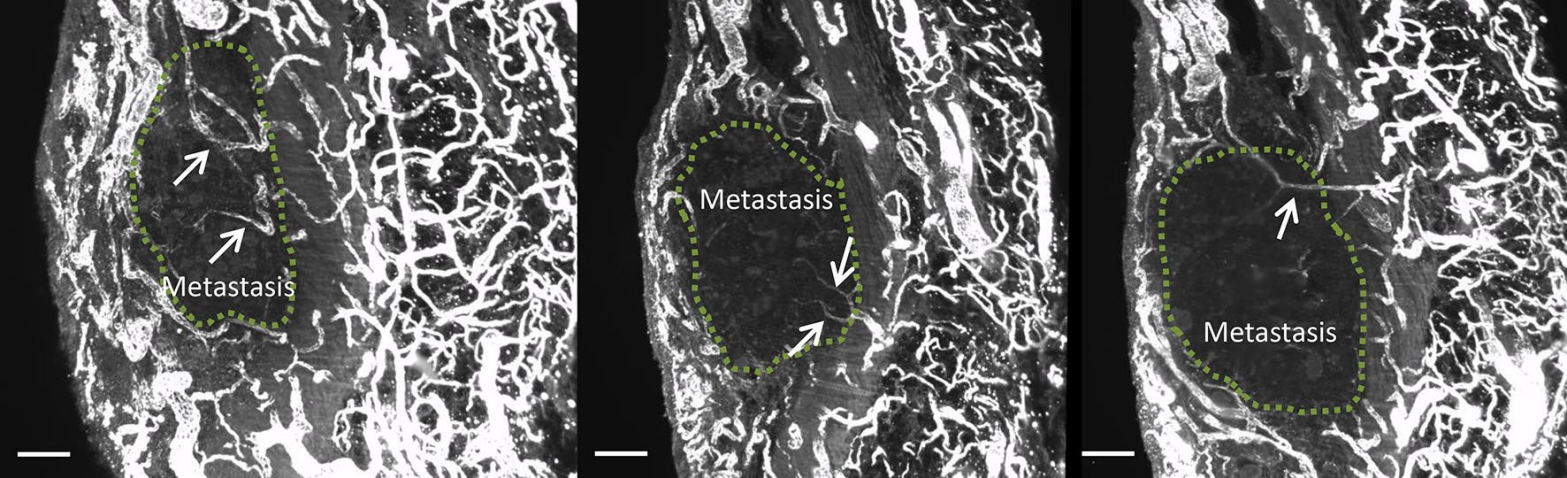
Light sheet fluorescence microscopy images of a peritoneal metastasis with CD31 staining. Arrows indicate vessels that originate from the submesothelial stroma, pass the elastic lamina, and invade the metastasis. Green dots indicate the boundary of the metastasis. Bar = 200 µm

One of the imaged EOC peritoneal metastases was biopsied, cleared and immunohistochemically stained for CD31, and subsequently analyzed with light sheet fluorescence microscopy. Figure 5 shows the 3D image of the metastasis located in the submesothelial stroma of the peritoneum. The metastasis distorted the shape of the peritoneal elastic lamina (PEL), but did not penetrate it. However, as shown in Fig. 6, capillaries from the submesothelial regions underneath the PEL were continuous with the capillaries of the metastasis. In other words, vascular sprouts were able to penetrate the PEL during angiogenesis, but EOC cells were not.

## Discussion

The results of our study suggest that (1) the perfusion of the microvasculature within peritoneal metastases of EOC is poor, (2) HIF-1α and VEGF expression are present in peritoneal metastases, (3) the microvascular networks of the peritoneum and EOC metastases are continuous through all peritoneal layers and (4) the intra-abdominal presence of EOC and/or PC does not affect the microvasculature in other unaffected regions of the peritoneum.

Tumor vasculature is known to differ widely between tumor types and to our knowledge, this is the first time that angioarchitecture and abnormalities in blood flow were studied and quantified in vivo in metastases of EOC [33]. The perfusion of the microvasculature in peritoneal metastases of EOC is most likely limited by the disorganized network of aberrant vascular structures [34]. Other theoretical explanations for the abnormal blood flow are the observed high levels of VEGF. High levels of VEGF can result in permeable and leaky vessels [8], which may further compromise perfusion. Since cancer and hemostasis are strongly related, thrombi in the microvasculature may also limit blood flow [35, 36]. In the current study, we have observed vessels with absent flow in which individual erythrocytes could not be discriminated, which is possibly caused by the presence of thrombi (Fig. 1C, D).

Our findings of the poor perfusion of the microvasculature in peritoneal metastases provide a possible explanation for the high peritoneal recurrence rate of EOC after IV-administered platinum-containing chemotherapy, which may be caused by ineffective delivery of insufficient concentrations of chemotherapy to peritoneal metastases [37]. It is tempting to speculate that the findings of our current study also partially explain the efficacy of (hyperthermic) intraperitoneal (IP) chemotherapy (HIPEC). It has been shown that (H)IPEC reduces the chance of recurrence and increases 5-year survival rates in patients with stage III EOC [38, 39]. During this procedure, the peritoneum is exposed to heated (41 °C) chemotherapy for 1.5 h. In case of poorly perfused microvasculature, the restricted blood flow may be less able to conduct heat and chemotherapy resulting in prolonged or more intense exposure in peritoneal metastases. The direct toxic effects of hyperthermia may be increased in this way, which is further intensified by the synergistic effect of chemotherapy [39].

We also hypothesized that a poorly perfused microvasculature in metastases may limit the exchange of oxygen, carbon dioxide and nutrients between the tissue and the vascular compartment, thus limiting expansive and invasive growth of EOC metastases into the peritoneum [40]. Expression of HIF-1α suggests that metastases are in fact hypoxic. Oxygen delivery to tissue primarily depends on the microvasculature and occurs through the hemodynamic principles convection and diffusion. Convection is quantified by flow, and diffusion is quantified by the spatial arrangement of capillaries. The relatively high TVD but low perfusion parameters suggest that hypoxia in metastases is a convection/flow problem, rather than a diffusion/TVD problem [40].

Three dimensional imaging demonstrated vascular continuity between the vascular networks of the submesothelial stroma and the peritoneal metastasis, across the PEL. The metastasis seemed to distort the PEL rather than penetrate it (Figs. 5, 6), which is in line with other studies that have demonstrated that some metastases do not seem capable of penetrating the PEL, whereas other metastases do penetrate it [6]. The findings of the current study suggest that blood vessel sprouts and in particular the tip cells of these sprouts invade the PEL. Proteolysis is needed for tissue invasion and tip cells are well equipped for these purposes [41]. Elastin is particularly difficult to hydrolyze, and only a few proteases are able to do this [42]. Apparently, angiogenesis through the PEL is possible, whereas invasion of the metastases through the PEL is more difficult.

Finally, an important finding of our study was that the presence of EOC does not affect the peritoneal vasculature in general (Fig. 2). It is known that tumors can modify the microenvironment of the prospective metastatic site, in this case the peritoneum, to pre-create an environment more favorable for tumor survival and growth [43, 44]. With IDF imaging, however, we did not observe an altered or increased vasculature in unaffected peritoneum, regardless the intra-abdominal presence of EOC or peritoneal metastases elsewhere. This does not exclude preparation of the microenvironment by immunologic changes at a cellular level.

### Strengths and limitations

This is the first study to assess and quantify the perfusion of the microvasculature in peritoneal metastases of EOC. In vivo imaging provided additional and crucial information, which cannot be obtained with the use of immunohistochemistry. Three-dimensional whole tumor imaging provided additional information on vascular structures connecting the vascular networks of the peritoneum and peritoneal metastases. The findings of this study generated hypotheses regarding pathophysiology and treatment of peritoneal carcinomatosis of EOC.

Limitations of this study include the limited focus depth of the CytoCam (max 300 µm), which can limit microvascular assessment of deeper vascular structures. However, the peritoneum is thin (approximately 50 µm in healthy subjects) and peritoneal metastases are small, thus very suitable for assessment with the CytoCam. Larger metastases may not allow full-depth assessment of their microvasculature. In these larger structures, it is likely that only the more superficial blood vessels are imaged. 3D imaging overcomes the limited focus depth, but provides no information on vessel perfusion. Another limitation of 3D imaging is that it requires clearing of the tissue, which is difficult in tumors. A limitation of CD31 staining is that endothelial cells are detected, also those which may not have formed capillaries yet. IDF imaging overcomes this limitation, because in the case of IDF imaging erythrocytes are visualized, which can only be present when there is flow, meaning that capillary formation must have occurred. It cannot be excluded that part of the observed effects is caused by neo-adjuvant chemotherapy. However, inter-participant differences of microvascular parameters did not occur and findings were consistent, irrespective of imaging during primary cytoreductive surgery or after neo-adjuvant chemotherapy. Also, the number of included participants was rather small, but because of the consistency of findings in the present study and our previous study [18], the differences are unlikely to be caused by coincidence.

The prevalence of cardiovascular risk factors in our study population was high, but comparable to the prevalence in the general population of similar age and gender [45]. Cardiovascular disease can affect the microvasculature, but this effect would also be present in normal peritoneum and not exclusively in metastases. Therefore, it is unlikely that differences between the microvasculature in metastases and normal peritoneum were caused by cardiovascular disease.

Finally, we visualized the vasculature in parietal peritoneum of the abdominal and pelvic wall. Although the microvascular anatomy is considered to be comparable in parietal and visceral peritoneum, future research is needed to investigate whether the vasculature in peritoneal metastases on the visceral peritoneum is similar. It is also interesting to investigate whether the microvasculature in peritoneal metastases of gastro-intestinal tumors is similar, or whether our findings are specific for metastases of EOC. In addition, it should be investigated whether HIPEC treatment affects the peritoneal microvasculature and whether microvascular parameters of the peritoneum correlate with course of disease and response to treatment of PC.

## Conclusion

We demonstrated that the perfusion of the microvasculature in peritoneal metastases of EOC is limited. Hypoperfusion may limit the therapeutic efficacy of IV chemotherapy and affect the behavior of EOC metastases on the peritoneum. Future studies should investigate whether microvascular parameters of the peritoneum correlate with disease outcome and response to therapy.

## Electronic supplementary material

Below is the link to the electronic supplementary material.

Online Resource 1 Four video clips from CytoCam – incident dark field imaging. First two videos show the microvasculature in peritoneum with macroscopically normal aspect. The blood flow was continuous. The third and fourth video show the microcirculation within a peritoneal metastasis of EOC. The blood flow was either sluggish or absent. (AVI 53324 kb)

## Abbreviations

AVI: Audio video interleave
CD31: Cluster of differentiation 31
DCM: Dichloromethane
DMSO: Dimethyl sulfoxide
EOC: Epithelial ovarian cancer
HGSC: High grade serous carcinoma
HIF-1α: Hypoxia inducible factor 1α
HIPEC: Hyperthermic intraperitoneal chemotherapy
IDF: Incident dark field
IP: Intraperitoneal
IV: Intravenous
LEDs: Light emitting diodes
PAX8: Paired box gene 8
PBS: Phosphate-buffered saline
PC: Peritoneal carcinomatosis
PEL: Peritoneal elastic lamina
PPV: Proportion of perfused vessels
PTwH: Phosphate-buffered saline/Tween with heparin
PVD: Perfused vessel density
RT: Room temperature
TBS: Tris-buffered saline
TVD: Total vessel density
VEGF: Vascular endothelial growth factor
